# Bacterial Vaccine Therapy

**Published:** 1914-03

**Authors:** 


Bacterial Vaccine Therapy.—Treatment of infectious diseases with preparations derived from corresponding micro-organisms is unquestionably growing in favor. Not only do the bacterial vaccines (or bacterins) seem destined to a permanent place in therapeutics, but their field of applicability is constantly broadening. Proof of this is seen in the growing list of these products announced by Parke, Davis & Co., no less than nineteen of the vaccines now being offered to the profession.

There are a number of reasons for the favor which is being accorded to the bacterial vaccines. In the first place these products are in consonance with the scientific trend of present-day medication. They are being used with a gratifying measure of success. The way in which they are marketed (sterile solutions in hermetically sealed bulbs and in graduated syringes, ready for injection) appeals to the modern medical man, since it assures both safety and convenience. The moderate prices at which they may be purchased also tend to give them vogue.

---

Fracture by slight or indirect violence suggests the possibility of some disease of the bone; so does non-union.—*American Journal of Surgery.*

